# Functional analysis of the glutathione S‐transferases from *Thinopyrum* and its derivatives on wheat Fusarium head blight resistance

**DOI:** 10.1111/pbi.14021

**Published:** 2023-02-10

**Authors:** Xianrui Guo, Qinghua Shi, Mian Wang, Jing Yuan, Jing Zhang, Jing Wang, Yang Liu, Handong Su, Zhen Wang, Jinbang Li, Cheng Liu, Xingguo Ye, Fangpu Han

**Affiliations:** ^1^ State Key Laboratory of Plant Cell and Chromosome Engineering Institute of Genetics and Developmental Biology, Innovation Academy for Seed Design, Chinese Academy of Sciences Beijing China; ^2^ Nanyang Academy of Agricultural Sciences Nanyang China; ^3^ Crop Research Institute Shandong Academy of Agricultural Sciences Jinan China; ^4^ Institute of Crop Sciences Chinese Academy of Agricultural Sciences Beijing China

**Keywords:** *Thinopyrum*, Fusarium head blight, Translocation line, *Fhb7*, glutathione S‐transferases

Fusarium head blight (FHB) is one of the devastating diseases for wheat production worldwide, which causes significant yield losses and reduces grain quality because of mycotoxins contamination in wheat grains. As wheat relatives, *Thinopyrum elongatum* and *Th. ponticum* are important genetic resources that can be used to improve wheat FHB resistance. Using recombinant inbred lines derived from a cross between two Thatcher‐*Th. ponticum* substitution lines, K11463 (7E1/7D) and K2620 (7E2/7D), the major FHB resistance locus *Fhb7* was mapped to the very distal region of the long arm of chromosome 7E2 (Guo *et al*., 2015). Wang *et al*. (2020) sequenced the genome of *Th. elongatum* and cloned the glutathione S‐transferase‐encoding *Fhb7* by genetic mapping. Relying on the recombination between *Th. elongatum* chromosome 7E and *Th. ponticum* chromosome 7E1, a resistant gene *Fhb‐7EL* for FHB resistance was located to the long arm of 7E (Ceoloni *et al*., 2017).

To transfer the resistant gene *Fhb‐7EL* to common wheat, hundreds of wheat‐*Th. elongatum* translocation lines were developed by irradiating the pollen of the wheat‐*Th. elongatum* addition line Chinese Spring (CS)‐7EL at anthesis, among which Zhongke 1878 proved to carry an approximately 100 Mb 7EL chromatin on chromosome 6DL (Figure 1a, Figure S1, Appendix S1). After backcrossing Zhongke 1878 with the highly susceptible variety Jimai 22 for six generations, FHB resistance evaluation showed that the translocated chromosome could significantly increase the FHB resistance of Jimai 22 to the level of Sumai 3 by decreasing the number of diseased spikelets from 13.43 to 1.43 (Figure 1b,c).

**Figure 1:**
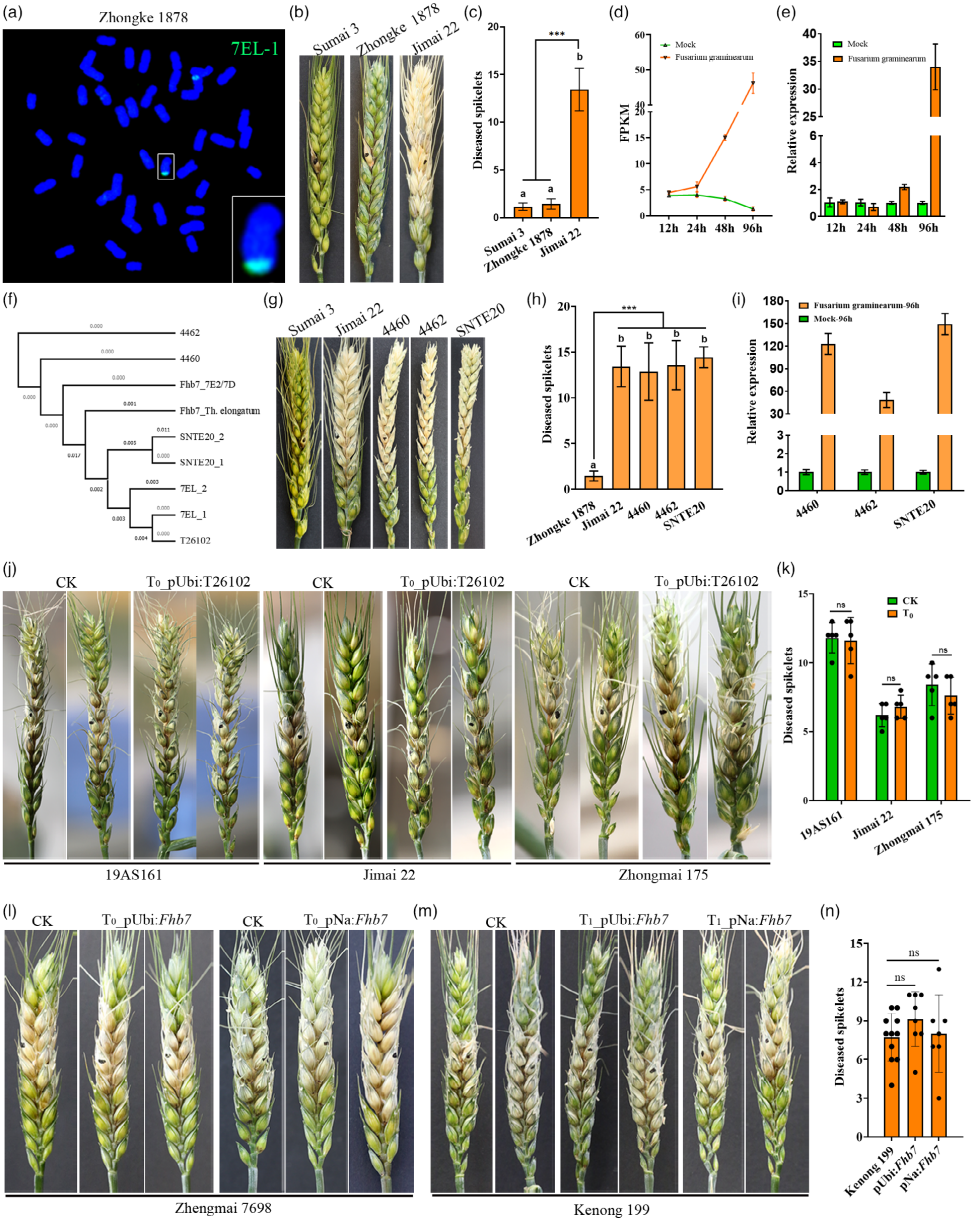
Functional analysis of the glutathione S‐transferases on FHB resistance. (a) Cytological analysis on the line Zhongke 1878. Alien chromatin was detected by using the green probe 7EL‐1. (b, c) FHB resistance evaluation on Zhongke 1878. The diseased spikes were photographed (b) and the number of diseased spikelets was calculated (c) at 21 days after inoculation with *Fusarium* species. (d, e) Expression pattern of T26102 in Zhongke 1878 measured by transcriptomic data (d) and qRT‐PCR (e). (f) Sequence comparisons of *Fhb7* homologues among wheat‐*Thinopyrum* derivatives. ‘1’ and ‘2’ indicate two different homologues in lines CS‐7EL and SNTE20. (g, h) FHB resistance evaluation on wheat‐*Thinopyrum* derivatives. The diseased spikes were photographed (g) and the number of diseased spikelets was calculated (h) at 21 days after inoculation with *Fusarium* species. (i) Expression analysis of T26102 96 h after inoculation with *Fusarium* species in wheat‐*Thinopyrum* derivatives. (j–n) FHB resistance evaluation on the transgenic lines with *Fhb7* homologues. The diseased spikes were photographed and the number of diseased spikelets was calculated at 7 days after inoculation with *Fusarium* species. pUbi:*Fhb7* indicated that *Fhb7* was driven by the *ubiquitin* promoter. pNa:*Fhb7* indicated that *Fhb7* was driven by the native promoter. (j, k) FHB resistance evaluation on the control (CK) and T_0_ transgenic lines overexpressing T26102. (l) FHB resistance evaluation on the T_0_ transgenic lines expressing *Fhb7* on the background Zhengmai 7698. (m, n) FHB resistance evaluation on the T_1_ transgenic lines expressing *Fhb7* on the background Kenong 199. ***P < 0.001, ns, *P* > 0.05.

To explore the nature of the FHB resistance gene, we inoculated the spikes of the line Zhongke 1878 with *F. graminearum* and performed single‐molecule real‐time isoform sequencing after 96 h. Removing the transcripts derived from wheat and *Fusarium* species by blasting wheat reference genome and nucleotide database on NCBI, 25 transcripts were identified derived from alien chromatin by PCR in Zhongke 1878 (Figure S2, Tables S1 and S2). To study the mechanisms of FHB resistance in the line Zhongke 1878, next‐generation sequencing‐based transcriptomic analysis was performed on these 25 transcripts. Annotated as a GST protein, the expression of the transcript T26102 was significantly increased 48 h after inoculation with *F. graminearum* (Figure 1d,e, Table S1).

To illustrate the association between T26102 and FHB resistance, the distribution of T26102 was checked in a series of wheat‐*Thinopyrum* derivatives. The homologue of T26102 was not only detected in wheat‐*Th. ponticum* amphiploid SNTE20 but also in the wheat‐*Th. ponticum* translocation lines 4460 and 4462 (Figure 1f, Figures S3, S4). After sequencing the amplified product, two different T26102 homologues were discovered in lines CS‐7EL, Zhongke 1878 and SNTE20 respectively (Figure 1f, Figure S4). Although SNTE20, 4460 and 4462 were proven to carry the GST‐encoding *Fhb7* homologues, all three lines were identified as susceptible to FHB as well as the susceptible control Jimai 22 (Figure 1g,h). Expression analysis revealed that *Fhb7* homologues were induced in lines 4460, 4462 and SNTE20 after inoculating with *F. graminearum* (Figure 1i). Similar results were also reported in the wheat‐*Th. ponticum* partial amphiploid SNTE122 and translocation line TNT‐B (Guo *et al*., 2022). More puzzling was that the *Fhb7* homologue and its promoter shared by 4460 and 4462 were identical to the one in the wheat‐*Th. ponticum* substitution line 7E2/7D used as the resistant parent to map *Fhb7* (Figure 1f, Figures S4, S5). All these results casted our doubt on the FHB‐resistant function of the GSTs.

To verify the FHB resistance function of T26102, we transformed the overexpression vector pUbi:T26102 into three common wheat accessions 19AS161, Jimai 22 and Zhongmai 175. The transgenic positive wheat plants overexpressing T26102 were used for FHB resistance evaluation (Figure S6). A few bleached spikelets were observed on all spikes of both wild types and T_0_ transgenic plants 7 days after inoculation with *F. graminearum* (Figure 1j). Statistical analysis was performed between the wild type and the transgenic plants; no difference was discovered between them (Figure 1k). To rule out the effect of amino acid variation on the function of T26102, we also expressed the GST‐encoding *Fhb7* under the *ubiquitin* promoter and the same native promoter as reported by Wang *et al*. (2020) in common wheat varieties Zhengmai 7698 and Kenong 199. Regardless of the vector driven by the *ubiquitin* promoter or the native promoter, nearly half the inoculated spikes bleached in the Zhengmai 7698 transgenic plants expressing *Fhb7* (Figure 1l). Except for the FHB evaluation on the T_0_ generation, the T_1_ transgenic plants on Kenong 199 background were chosen to verify the function of *Fhb7*. With obvious bleached spikelets on the inoculated spikes, no statistical difference in FHB resistance was discovered between the T_1_ transgenic plants and the control Kenong 199 (Figure 1m,n). These results suggested the GST‐encoding *Fhb7* also failed to confer wheat FHB resistance. All these results suggested that GSTs from *Thinopyrum*, including the GST‐encoding *Fhb7* and its homologues, were not decisive for FHB resistance.

## Accession number

All transcriptomic raw reads for the translocation line Zhongke 1878 are available from the NCBI BioProject under accession number PRJNA720120.

## Conflict of interest

The authors declare no conflict of interest.

## Author contribution

F. H. and X. Y. conceived the study. X. G., Q. S. and M. W. conducted the experiment. J. Y., Y. L., J. Z. and J. W. participated in vector construction. H. S. contributed bioinformatics analysis. Z. W., J. L. and C. L. helped FHB resistance evaluation. X. Y. contributed the transgene experiment. X. G. and F. H wrote the manuscript.

## Supporting information


**Appendix S1** Methods.

**Figure S1** Coverage analysis on the Zhongke.
**Figure S2** Twenty‐five specific alien transcripts identified by PCR in Zhongke 1878.
**Figure S3** Cytological analysis on wheat‐*Thinopyrum* derivatives carrying *Fhb7* homologs.
**Figure S4** Protein sequence alignments of *Fhb7* homologs in wheat‐*Thinopyrum*, derivatives.
**Figure S5** Promoter sequence alignment of *Fhb7* homologs.
**Figure S6** Expression analysis of *Fhb7* homolog in transgenic wheat plants.

**Table S1** The sequences and function annotation of twenty‐five specific transcripts in line Zhongke 1878.
**Table S2** Primers used in this study.
