# Supplementary material for: Functional analysis of the glutathione S‐transferases from Thinopyrum and its derivatives on wheat Fusarium head blight resistance
Source: Plant Biotechnol J. 2023 Feb 10;21(6):1091–3. doi: 10.1111/pbi.14021 (PMC10214746; doi:10.1111/pbi.14021)
Supplement: Supplementary file 1 — Appendix S1 Methods. [file PBI-21-1091-s001.docx]

Supplemental Methods for

**Functional analysis of the glutathione S-transferases from *Thinopyrum* and its derivatives on wheat Fusarium head blight resistance**

**Methods**

**Nucleic acid extraction**

Genomic DNA of the seedlings was extracted with Plant Genomic DNA Kit (TianGen, Beijing, China). Total RNA from leaves and spikes was extracted using TRIzol^®^ Reagent (Thermo Fisher Scientific, Shanghai, China) and the first-strand cDNA synthesis from the total RNA was performed by using the FastKing RT kit (with gDNase) (TianGen, Beijing, China). Extraction of plasmid DNA was performed by using the TIANprep Mini Plasmid Kit (TianGen, Beijing, China).

**Fluorescence *in situ* hybridization (FISH)**

The translocation lines were screened by FISH according to previously reported methods ([Guo and Han, 2014](#_ENREF_1)). The seeds harvested were germinated on moist filter paper in a petri dish at room temperature for 2-3 days. The roots were cut from the seedlings and then placed in nitrous oxide for 2 hours. Subsequently the roots were fixed in 90% acetic acid for 5 minutes and then washed three times by sterile water. Chromosome spreads preparation was performed as previously described ([Kato et al., 2004](#_ENREF_3)). 7EL-1 was obtained by DOP-PCR from the 7EL library constructed by chromosome microdissection, and was specific for the genome of *Th. elongatum* and *Th. ponticum*. The probes were labeled using the nick translation method ([Han et al., 2006](#_ENREF_2)). 7EL-1 was labelled with Alexa Fluor-488-5-dUTP. The centromeric retrotransposon of wheat clone 6C6 and pAs1 were labeled with Texas-red-5-dCTP.

**Fusarium head blight resistance evaluation**

During the period of 2017-2019 in greenhouse, FHB evaluations in greenhouse were performed by using the single spikelet inoculation method ([Lulin et al., 2010](#_ENREF_4)). The temperature was controlled at 25~30℃. Equal mixing three pathogenic *F. graminearum* strains (Fg16-2, Fg16-5 and Fg16-11) and one *Fusarium asiaticum* strain (Fa301) in Mung bean broth produced fungal spores. For convenience, we referred to the four mixed species as *F. graminearum*. Approximately 20 μL of *F. graminearum* fungal suspension (1× 10^6^ conidia/ml) was injected into the central spikelet at early flowering stage. The inoculated spikes were covered with a plastic bag for 2 days to keep moist for fungal infection. The percentage of diseased spikelets for each spike was recorded at 7, 10 and 21 days after inoculation. The wheat varieties Jimai 22 and Sumai 3 were used as susceptible and resistant control, respectively.

**Genome resequencing and coverage analysis**

A combined reference genome is consisting of IWGSC Chinese Spring reference genome v1.0 and the genomic sequence of chromosome 7E extracted from *Th. elongatum* reference genome (D-3458). Raw reads were first processed with Trimmomatic (v.0.36) (with parameters: SLIDINGWINDOW:5:20 LEADING:5 TRAILING:5 MINLEN:25). Cleaned reads were aligned to the combined reference genome with BWA mem (v.0.7.13-r1126). Alignments were compressed, sorted, filtered (remove mapping quality less than 20) and indexed by using SAMtools (v.1.9). Duplicate alignments were removed with Picard (v.2.25.6). The means of read depth within per 100kb window were calculated using Mosdepth (v.0.2.6) with default parameters. The chromosome translocation points were identified in IGV (v.2.12.0).

**RNA sequencing and screening transcripts induced by *F. graminearum***

To explore the resistance gene for FHB, the spikes of the translocation line Zhongke 1878 were sampled for RNA sequencing after inoculating with *F. graminearum*. Three spikelets around the inoculated one from at least three spikes of different plants were collected at 12, 24, 48, and 96 h post inoculation and grounded in liquid nitrogen for Illumina RNA-Seq. As lesions were observed on the glumes at 96 h post inoculation, the sample at 96 h was selected for full-length transcriptome sequencing. All transcriptomic raw reads for the translocation line Zhongke 1878 are available from the NCBI BioProject under accession number PRJNA720120. Firstly, we aligned the sequenced transcripts on the Chinese Spring reference genome (IWGSC Chinese Spring reference genome v1.0) by using the software GMAP (with parameters: -min-trimmed-coverage 0.9 -min-identity 0.85). To remove the transcripts derived from the inoculated *F. graminearum* isolates, the unmapped transcripts were blasted against the nucleotide database on the National Center for Biotechnology Information (NCBI). In order to confirm their origin, one to three pairs of primers were designed for the remaining transcripts. Polymerase chain reaction was carried out using the genome DNA of the wheat-*Th. elongatum* addition line CS-7EL, Chinese Spring, Zhongke 1878 and Jimai 22. The functions of the transcripts specific for Zhongke 1878 were annotated by using Blastx on the NCBI. Data analysis was performed by employing HISAT2 and StringTie according to the previous report ([Pertea et al., 2016](#_ENREF_5)). The FPKM value from StringTie was used to measure the expression level.

**Detection and expression analysis of *Fhb7* homologs**

The fragment of T26102 was amplified by using the primer set of 26102FA1 and 26102CDS-R (Table S2). The fragments amplified from different species were cloned onto the *pEASY*-T1 simple cloning vector (TransGen, Beijing, China) for sequencing. First-strand cDNA synthesis from the total RNA was performed by using the FastKing RT kit (with gDNase) (TianGen, Beijing). The expression analyses were performed using the primer set 26102RT-F and 26102RT-R. The gene *actin* was used as an internal standard. The relative expression of *Fhb7* homologs was calculated by the 2 ^–ΔΔCT^ method.

**Genetic transformation of *Fhb7* homologs mediated by Agrobacterium**

The 846 bp CDS of T26102 was amplified from the genomic DNA of the translocation line Zhongke 1878 and the GST-encoding *Fhb7* was cloned by performing point-mutagenesis PCR. Both CDSs were cloned into the MCS of the modified pWMB110 vector under the *ubiquitin* promoter by using the EasyGeno Assembly Cloning kit (TianGen, Beijing, China). For the GST-encoding *Fhb7*, the native promoter sequence was amplified from the translocation line 4460 and the recombinant sequence was cloned into the modified pWMB110 vector. All recombinant plasmids were separately transformed into *Agrobacterium* strain C58C1 (Zoman Biotech Co, Beijing). The *Agrobacterium*-mediated wheat transformation using the immature embryos of 19AS161, Zhongmai 175, Jimai 22, Zhengmai 7698 and Kenong 199 was carried out as previously described ([Wang et al., 2017](#_ENREF_6)). The positive T_0_ and T_1_ plants expressing the *Fhb7* or its homolog were used for FHB resistance evaluation.

Guo, X. and Han, F. (2014) Asymmetric epigenetic modification and elimination of rDNA sequences by polyploidization in wheat. *Plant Cell* **26**, 4311-4327.

Han, F., Lamb, J.C. and Birchler, J.A. (2006) High frequency of centromere inactivation resulting in stable dicentric chromosomes of maize. *Proc Natl Acad Sci U S A* **103**, 3238-3243.

Kato, A., Lamb, J.C. and Birchler, J.A. (2004) Chromosome painting using repetitive DNA sequences as probes for somatic chromosome identification in maize. *Proc Natl Acad Sci U S A* **101**, 13554-13559.

Lulin, M., Yi, S., Aizhong, C., Zengjun, Q., Liping, X., Peidu, C., Dajun, L. and Xiu, E.W. (2010) Molecular cloning and characterization of an up-regulated UDP-glucosyltransferase gene induced by DON from Triticum aestivum L. cv. Wangshuibai. *Mol. Biol. Rep.* **37**, 785-795.

Pertea, M., Kim, D., Pertea, G.M., Leek, J.T. and Salzberg, S.L. (2016) Transcript-level expression analysis of RNA-seq experiments with HISAT, StringTie and Ballgown. *Nat Protoc* **11**, 1650-1667.

Wang, K., Liu, H., Du, L. and Ye, X. (2017) Generation of marker-free transgenic hexaploid wheat via an Agrobacterium-mediated co-transformation strategy in commercial Chinese wheat varieties. *Plant Biotechnol. J.* **15**, 614-623.
