# Supplementary material for: Functional analysis of the glutathione S‐transferases from Thinopyrum and its derivatives on wheat Fusarium head blight resistance
Source: Plant Biotechnol J. 2023 Feb 10;21(6):1091–3. doi: 10.1111/pbi.14021 (PMC10214746; doi:10.1111/pbi.14021)
Supplement: Supplementary file 2 — Figure S1 Coverage analysis on the Zhongke. Figure S2 Twenty‐five specific alien transcripts identified by PCR in Zhongke 1878. Figure S3 Cytological analysis on wheat‐Thinopyrum derivatives carrying Fhb7 homologs. Figure S4 Protein sequence alignments of Fhb7 homologs in wheat‐Thinopyrum, derivatives. Figure S5 Promoter sequence alignment of Fhb7 homologs. Figure S6 Expression analysis of Fhb7 homolog in transgenic wheat plants. [file PBI-21-1091-s002.pptx]

## Slide 1
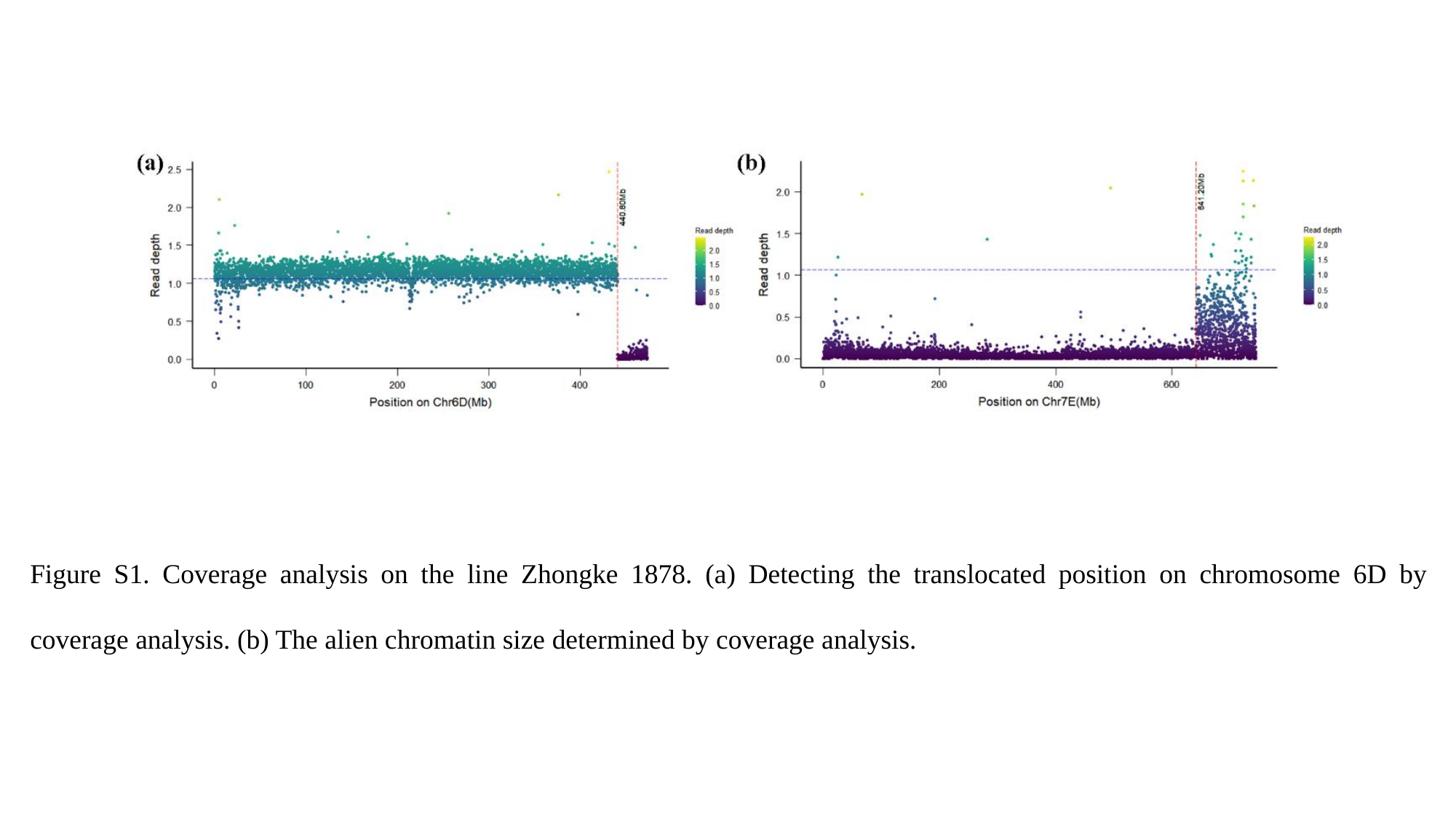

Figure S1. Coverage analysis on the line Zhongke 1878. (a) Detecting the translocated position on chromosome 6D by coverage analysis. (b) The alien chromatin size determined by coverage analysis.

## Slide 2
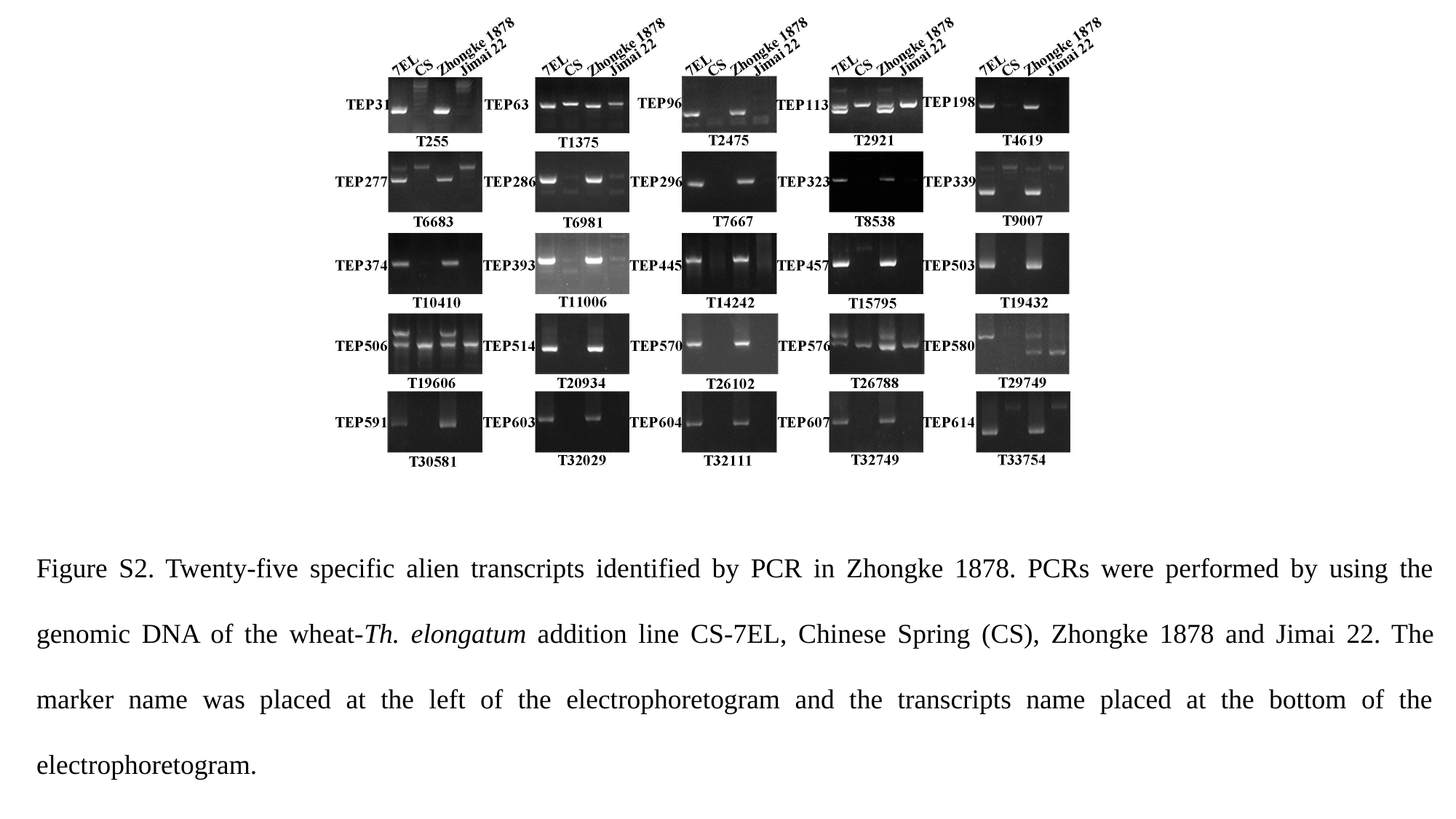

Figure S2. Twenty-five specific alien transcripts identified by PCR in Zhongke 1878. PCRs were performed by using the genomic DNA of the wheat-Th. elongatum addition line CS-7EL, Chinese Spring (CS), Zhongke 1878 and Jimai 22. The marker name was placed at the left of the electrophoretogram and the transcripts name placed at the bottom of the electrophoretogram.

## Slide 3
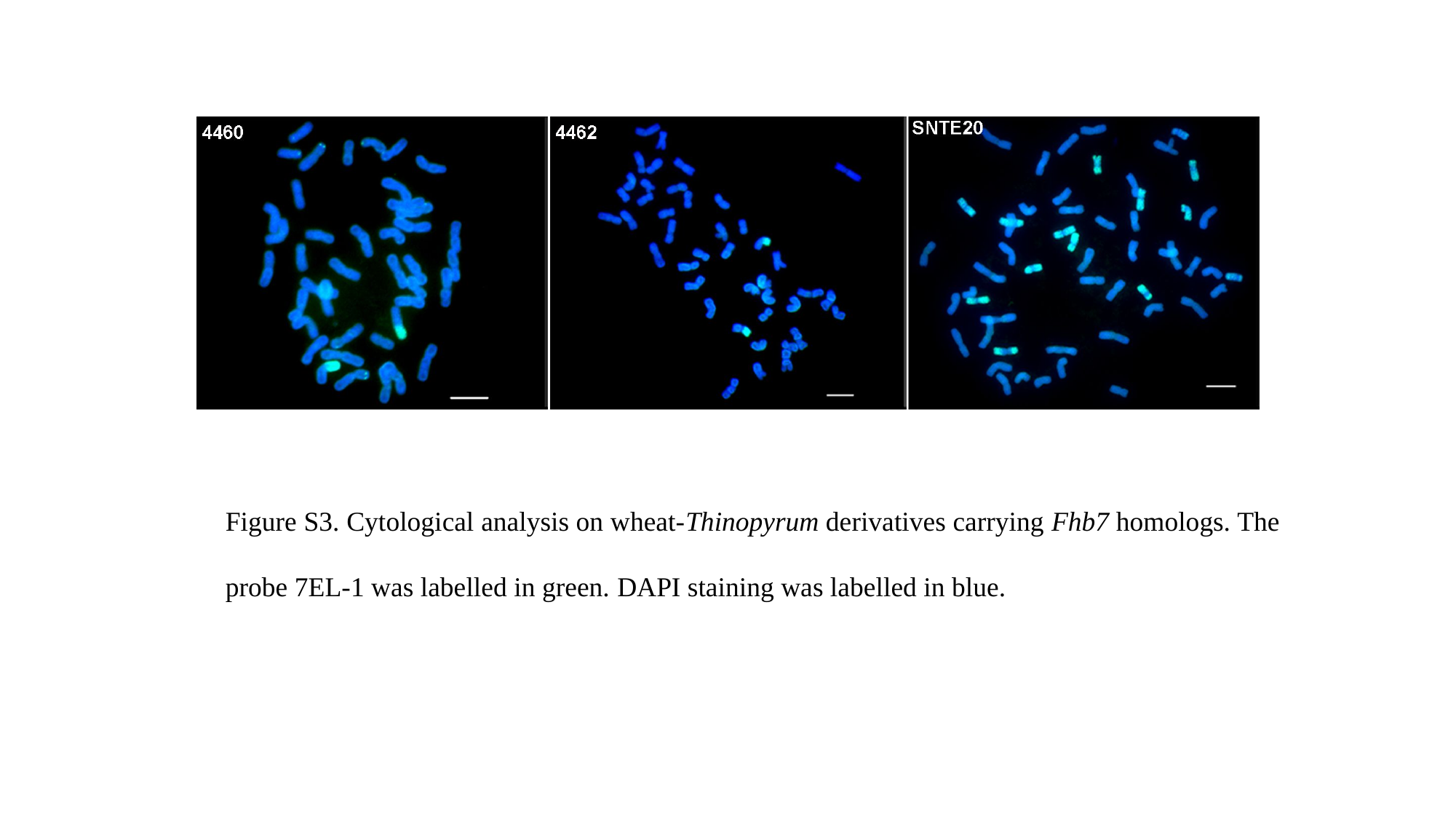

Figure S3. Cytological analysis on wheat-Thinopyrum derivatives carrying Fhb7 homologs. The probe 7EL-1 was labelled in green. DAPI staining was labelled in blue.

## Slide 4
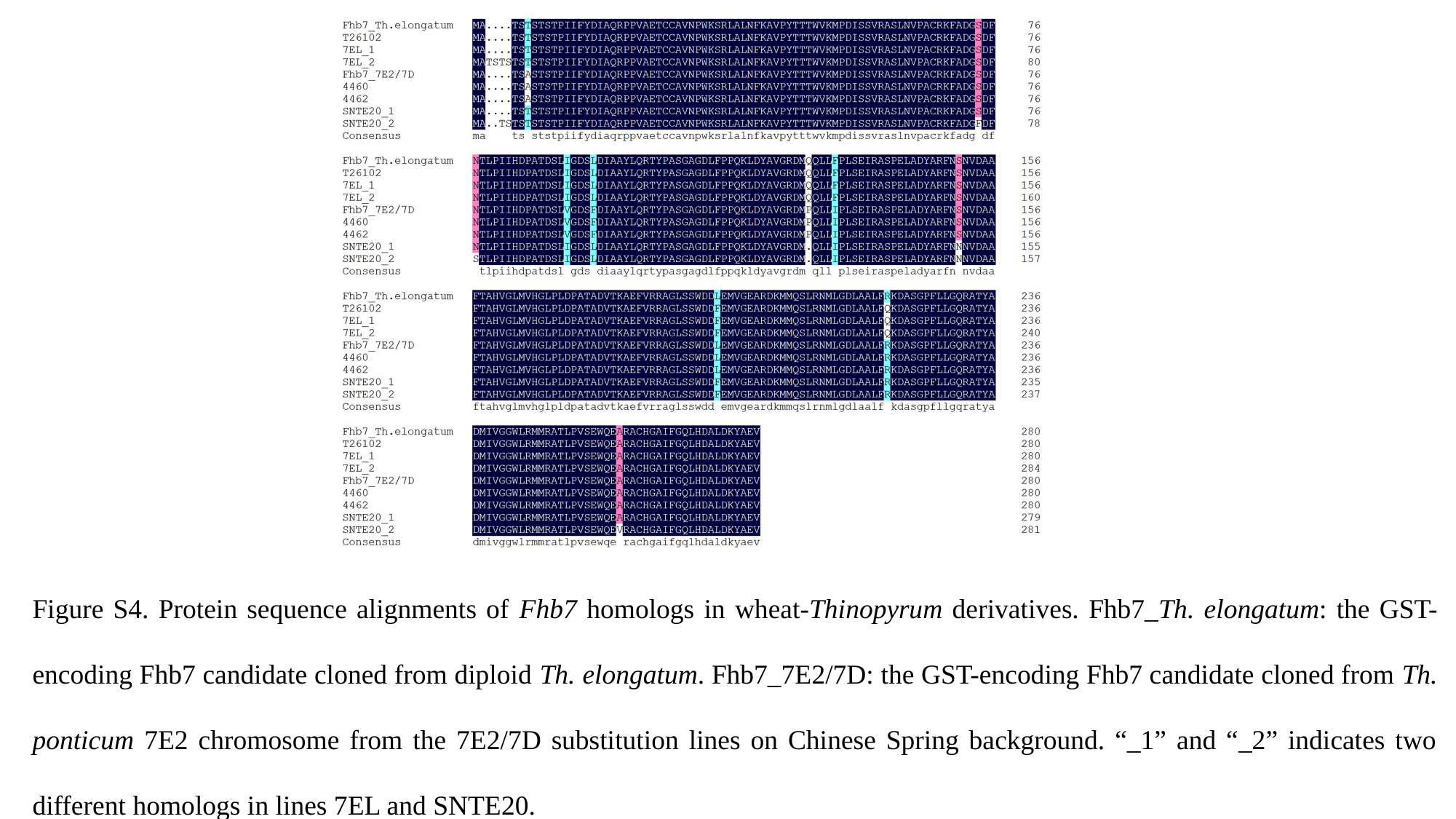

Figure S4. Protein sequence alignments of Fhb7 homologs in wheat-Thinopyrum derivatives. Fhb7_Th. elongatum: the GST-encoding Fhb7 candidate cloned from diploid Th. elongatum. Fhb7_7E2/7D: the GST-encoding Fhb7 candidate cloned from Th. ponticum 7E2 chromosome from the 7E2/7D substitution lines on Chinese Spring background. “_1” and “_2” indicates two different homologs in lines 7EL and SNTE20.

## Slide 5
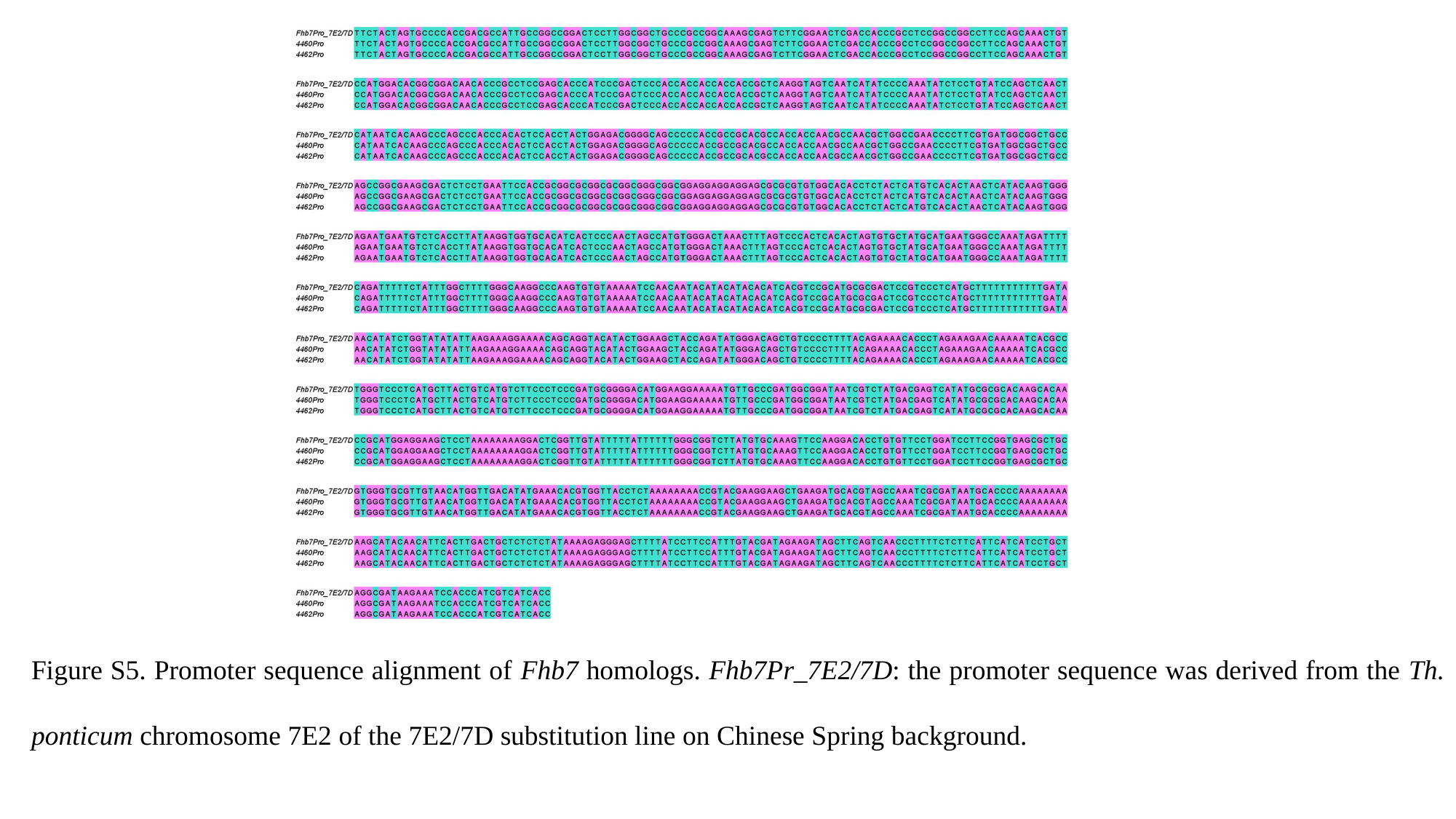

Figure S5. Promoter sequence alignment of Fhb7 homologs. Fhb7Pr_7E2/7D: the promoter sequence was derived from the Th. ponticum chromosome 7E2 of the 7E2/7D substitution line on Chinese Spring background.

## Slide 6
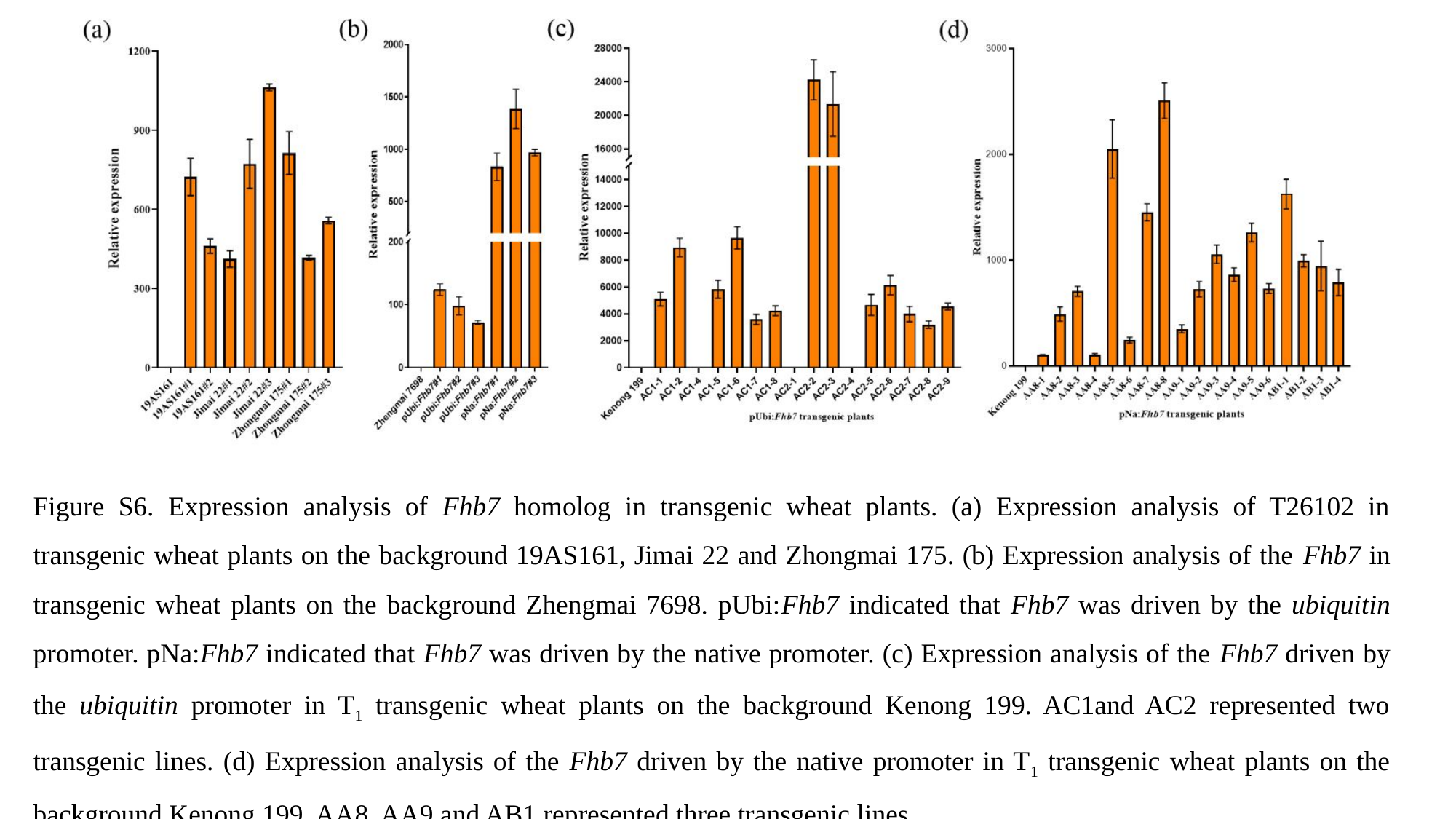

Figure S6. Expression analysis of Fhb7 homolog in transgenic wheat plants. (a) Expression analysis of T26102 in transgenic wheat plants on the background 19AS161, Jimai 22 and Zhongmai 175. (b) Expression analysis of the Fhb7 in transgenic wheat plants on the background Zhengmai 7698. pUbi:Fhb7 indicated that Fhb7 was driven by the ubiquitin promoter. pNa:Fhb7 indicated that Fhb7 was driven by the native promoter. (c) Expression analysis of the Fhb7 driven by the ubiquitin promoter in T1 transgenic wheat plants on the background Kenong 199. AC1and AC2 represented two transgenic lines. (d) Expression analysis of the Fhb7 driven by the native promoter in T1 transgenic wheat plants on the background Kenong 199. AA8, AA9 and AB1 represented three transgenic lines.
